# Interfacial contact stiffness of fractal rough surfaces

**DOI:** 10.1038/s41598-017-13314-2

**Published:** 2017-10-09

**Authors:** Dayi Zhang, Ying Xia, Fabrizio Scarpa, Jie Hong, Yanhong Ma

**Affiliations:** 1 School of Energy and Power Engineering, Beihang University, Beijing, 100191 P.R. China; 2 Bristol Composites Institute (ACCIS), University of Bristol, BS8 1TR Bristol, UK; 3 Dynamics and Control Research Group (DCRG), CAM School, University of Bristol, BS8 1TR Bristol, UK; 4 Collaborative Innovation Center of Advanced Aero-Engine, Beihang University, Beijing, 100083 P.R. China

## Abstract

In this work we describe a theoretical model that predicts the interfacial contact stiffness of fractal rough surfaces by considering the effects of elastic and plastic deformations of the fractal asperities. We also develop an original test rig that simulates dovetail joints for turbo machinery blades, which can fine tune the normal contact load existing between the contacting surfaces of the blade root. The interfacial contact stiffness is obtained through an inverse identification method in which finite element simulations are fitted to the experimental results. Excellent agreement is observed between the contact stiffness predicted by the theoretical model and by the analogous experimental results. We demonstrate that the contact stiffness is a power law function of the normal contact load with an exponent *α* within the whole range of fractal dimension *D*(1 < *D* < 2). We also show that for 1 < *D* < 1.5 the Pohrt-Popov behavior (*α* = 1/(3 − *D*)) is valid, however for 1.5 < *D* < 2, the exponent *α* is different and equal to 2(*D* − 1)/*D*. The diversity between the model developed in the work and the Pohrt-Popov one is explained in detail.

## Introduction

In the design of mechanical components the surface roughness plays a significant part. Roughness makes the actual contact area noticeably smaller than the nominal contact surface in a considerable number of relevant cases^1^. This fact is of tremendous practical importance since the size and shape of the real contact area affect a large number of physical properties relevant to mechanical engineering and electrical and heating conduction problems. The interfacial contact stiffness that frequently dominates the overall static and dynamic characteristics of mechanical systems is one of the most significant properties. Examples for which interfacial contact stiffness is prominent are constituted by friction dampers in jet engines^2,3^, rotor systems^4,5^, gears^6^, head-disk and brake interface^7^ and micro electro-mechanical systems (MEMS)^8^.

Interfacial contact stiffness is mainly estimated through experimental measurements. A variety of measurement technologies have been developed for the purpose during the last decades, such as the contact resonant frequency method^9^, the ultrasonic method^10^, the continuous stiffness measurement^11^, and the digital image correlation^12^. Several test rigs have also been designed for specific contact conditions, and some are suitable for high sliding velocities and small displacements^13,14^, while others are applicable for high loads, large displacements, and relatively low velocities^15^. A theoretical model is however still needed to predict the interfacial contact stiffness for the entire range of the design parameters of the mechanical component. The most widely used model is the Greenwood–Williamson (GW) statistical one^16^. The precision of the GW model is however limited, since the parameters used to describe the roughness strongly depend on the resolution of the roughness-measuring instrument. Works by Majumdar and Bhushan^17,18^ show however that a variety of surfaces with roughness are self-affine. Based on the representation of the surface as a fractal geometry, the scale-invariant fractal parameters can indeed characterize in an adequate manner the roughness at all length scales^19^. Since those seminal works, recent interest in the contact mechanics of areas with roughness has focused on the adoption of models dealing with fractal and self-affine rough surfaces. The most relevant study to this article is the contact stiffness model of plane machined joints based on fractal theory by Jiang^20^. This model assumes that the asperities are sufficiently widely spaced to be treated as resistances in parallel, however only elastic asperities are considered for the calculation of the contact stiffness.

In our work we present a generalized model of the contact stiffness between areas with roughness described in terms of fractal profiles. The model considers the whole interfaces represented by fractal surfaces, and both plastic and elastic asperities are included. We also describe the design and use of a test rig to simulate the dovetail joint of a turbo machinery blade under operating status. The rig can accurately measure the dependency of the 1^st^ resonant frequency of the blade with the normal contact load. To verify our model, the contact stiffness corresponding to the normal load is identified by correlating the experimental results to the simulation results based on the contact resonant frequency method.

## Results and Discussions

The contact between two surfaces with roughness can be modeled as an equivalent rugged surface contacting a smooth rigid plane, and with a large number of separate asperities of different sizes spreading over the irregular surface contacting the rigid plane. The contact stiffness *K*_*n*_ is the partial derivative of the normal load *P* versus the relative displacement *∆* of the rigid plane from the equivalent rough surface:

1 $${K}_{n}\,=\,\frac{\partial P}{\partial {\rm{\Delta }}}$$

**Figure 1:**
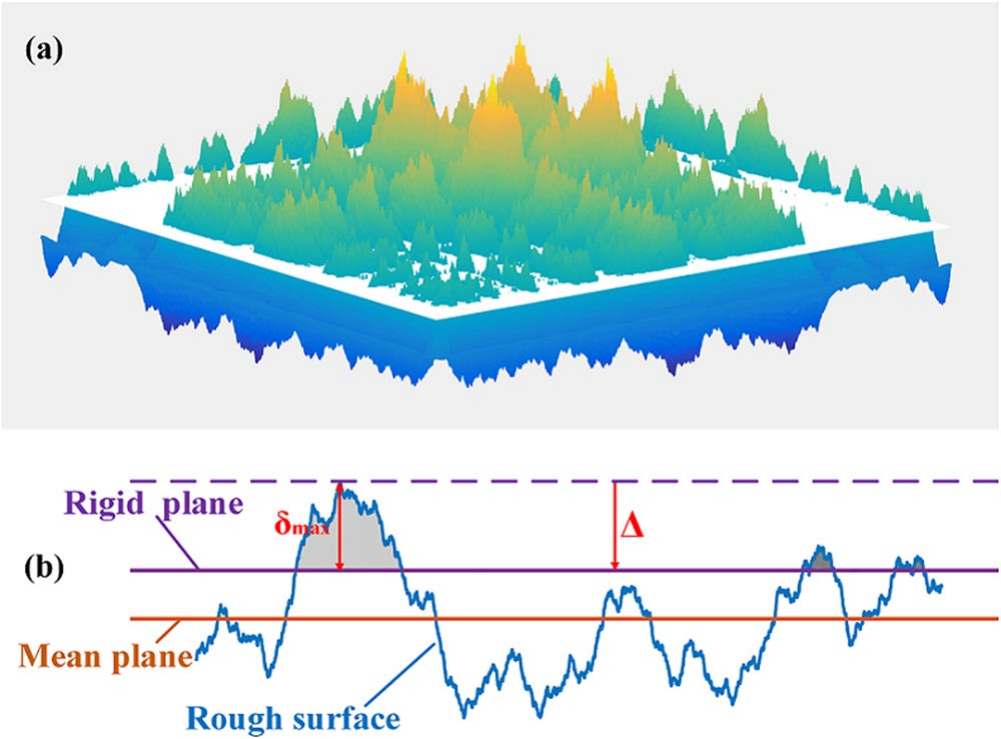
(**a**) One undeformed rough surface (in color) with one smooth rigid plane (white), and (**b**) the 2D section of the surfaces.

As illustrated in Fig. 1, we consider that the relative displacement *∆* of the rigid plane from the equivalent rugged surface is equal to the largest interference of a single asperity:

2 $${\rm{\Delta }}\,=\,{\delta }_{\max }$$

In the case of fractal rugged surfaces, the theoretical model representing the contact stiffness is obtained by associating equations (1 and 2) with the normal contact load between two fractal surfaces, as derived in^21^. When the truncated micro-contact area of the largest asperity ${a}_{l}^{^{\prime} }$ is smaller than the critical truncated micro-contact area ${a}_{c}^{^{\prime} }$, all the asperities that belong to the equivalent rough surface are plastically deformed. The interfacial contact stiffness is therefore described as:

3 $${K}_{n}\,=\,\frac{HD{\pi }^{(2-D)/2}}{{2}^{(2-D)}{(2-D)}^{2}{G}^{(D-1)}{(\mathrm{ln}\gamma )}^{1/2}}{({a}_{l}^{^{\prime} })}^{D/2}$$

When ${a}_{l}^{^{\prime} }$ is larger than ${a}_{c}^{^{\prime} }$, both plastic and elastic deformations exist and the interfacial contact stiffness is:

4 $$\begin{array}{rcl}{K}_{n} & = & \frac{4D(3-D)E\ast }{3\sqrt{2\pi }(3-2D)(2-D)}{({a}_{l}^{^{\prime} })}^{1/2}-\frac{4{D}^{2}E\ast }{3\sqrt{2\pi }(3-2D)(2-D)}\,{({a}_{l}^{^{\prime} })}^{(D-1)}{a}_{c}^{^{\prime} (3/2-D)}\\  &  & +\,\frac{H{D}^{2}{\pi }^{(2-D)/2}}{{2}^{(3-D)}{(2-D)}^{2}{G}^{(D-1)}{(\mathrm{ln}\gamma )}^{1/2}}\,{({a}_{l}^{^{\prime} })}^{(D-1)}{a}_{c}^{^{\prime} (1-D/2)}\,(1 < D < 2,D\ne 1.5)\\ {K}_{n} & = & \frac{4{E}^{\ast }}{\sqrt{2\pi }}{({a}_{l}^{^{\prime} })}^{1/2}+\frac{3{E}^{\ast }}{\sqrt{2\pi }}{({a}_{l}^{^{\prime} })}^{1/2}\,\mathrm{ln}\,({a}_{l}^{^{\prime} }/{a}_{c}^{^{\prime} })+\frac{9H{\pi }^{1/4}}{{2}^{3/2}{G}^{1/2}{(\mathrm{ln}\gamma )}^{1/2}}{({a}_{l}^{^{\prime} })}^{1/2}{a}_{c}^{^{\prime} 1/4}\,(D=1.5)\end{array}$$

In (4) *E* ^*^ and *H* represent the equivalent Young’s modulus and the hardness of the softer material of the mating surfaces. The parameter *D* constitutes the fractal dimension of the surface topography, *G* is the fractal roughness parameter while *γ* is a non-dimensional quantity related to the profile describing the fractal surface.

**Figure 2:**
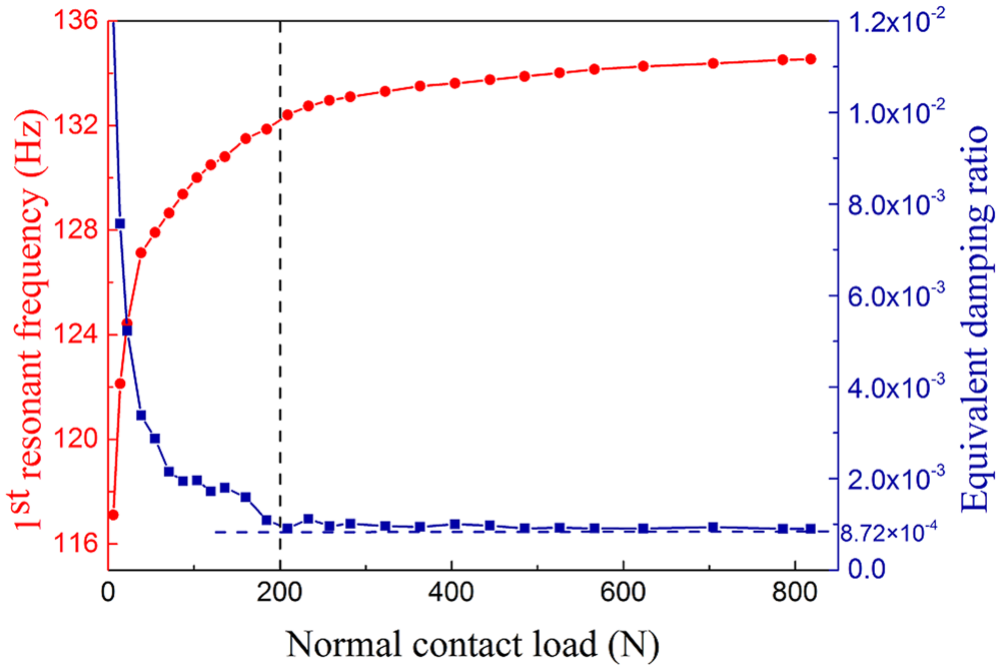
Variations of the resonant frequency and the equivalent damping ratio versus the normal contact load.

To verify the proposed theoretical model, we measure the 1^st^ frequency (global mode) of a blade-dovetail joint system from frequency response functions (FRF) under different normal contact loads. The resonant frequency is determined from the peak point of the FRF, while the equivalent modal damping ratio is determined using the half-power method. The measured damping ratio is $8.72\times {10}^{-4}$ when the contacting surfaces are glued. The dependencies of the resonant frequency and the equivalent damping ratio on the normal load are shown in Fig. 2. Partial slip occurs on the contacting interfaces when the normal contact load is less than 200 N. Beyond this force value, the interfaces appears to be in full stick. We can therefore reasonably neglect the presence of macroscopic effects in the mechanics of the contact when the surfaces are subjected to a full stick state.

**Figure 3:**
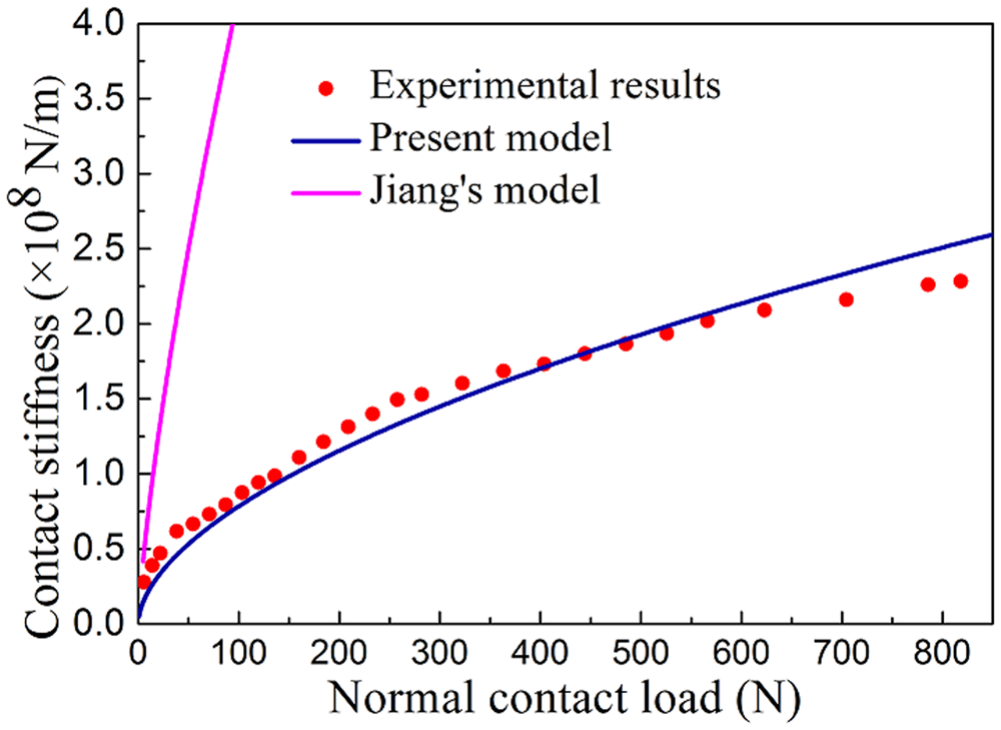
Dependency of the contact stiffness on the normal contact load.

The contact stiffness is experimentally measured by using an inverse identification method, which consists in fitting numerical results from a high-fidelity finite element model representing the blade, to the measured dynamic velocities measured for the 1^st^ resonant frequency. More details about the experiment and the identification are provided in the following Sections. The variation of the theoretical contact stiffness and the experimental results on the normal contact load are shown in Fig. 3. The contact stiffness calculated from Jiang’s model is also given as a further comparison. The coincidence between theoretical and experimental results indicates that the present model is more appropriate. The contact stiffness provided by the widely used Jiang’s model appears to be significantly larger than the one calculated from the present model and the experimental results.

The contact stiffness from Jiang’s model is calculated by integrating the stiffness of a single elastic asperity:

5 $${K}_{n}={\int }_{{a^{\prime} }_{c}}^{{a^{\prime} }_{l}}{k}_{n}^{e}\bullet n(a^{\prime} )da^{\prime} =\frac{4{E}^{\ast }D(3-D)}{3\sqrt{2\pi }(2-D)(D-1)}({a}_{l}^{^{\prime} D/2}{a}_{c}^{^{\prime} (1/2-D/2)}-{a}_{l}^{^{\prime} 1/2})$$

where $n(a^{\prime} )$ is the distribution density of the asperities with truncated micro-contact area $a^{\prime}$.

Jiang’s model assumes that each asperity behaves as an elastic spring, and the equivalent surface can be regarded as a series of springs in parallel, with ${a^{\prime} }_{c}\to 0$ leading to ${K}_{n}\to +\infty$. The stiffness of each single elastic asperity decreases for lower truncated micro contact area, however the density of the spring distribution would increase and result in the whole contact stiffness tending to ${K}_{n}\to \,+\,\infty$. Although the micro contact area ${a^{\prime} }_{c}$ is certainly not an infinitesimal, smaller asperities in this model tend nonetheless to contribute significantly to the whole contact stiffness. This assumed mechanistic behaviour is however quite different from the one exposed in recent papers^22^, for which the contact stiffness is dominated by the presence of larger asperities and it is insensitive to the behavior at the microscopic scale. We can therefore conclude that the integration form of the whole contact stiffness tends to overestimate the contribution provided by smaller asperities under the given distribution density, leading to significantly higher stiffness values than the experimental ones.

**Figure 4:**
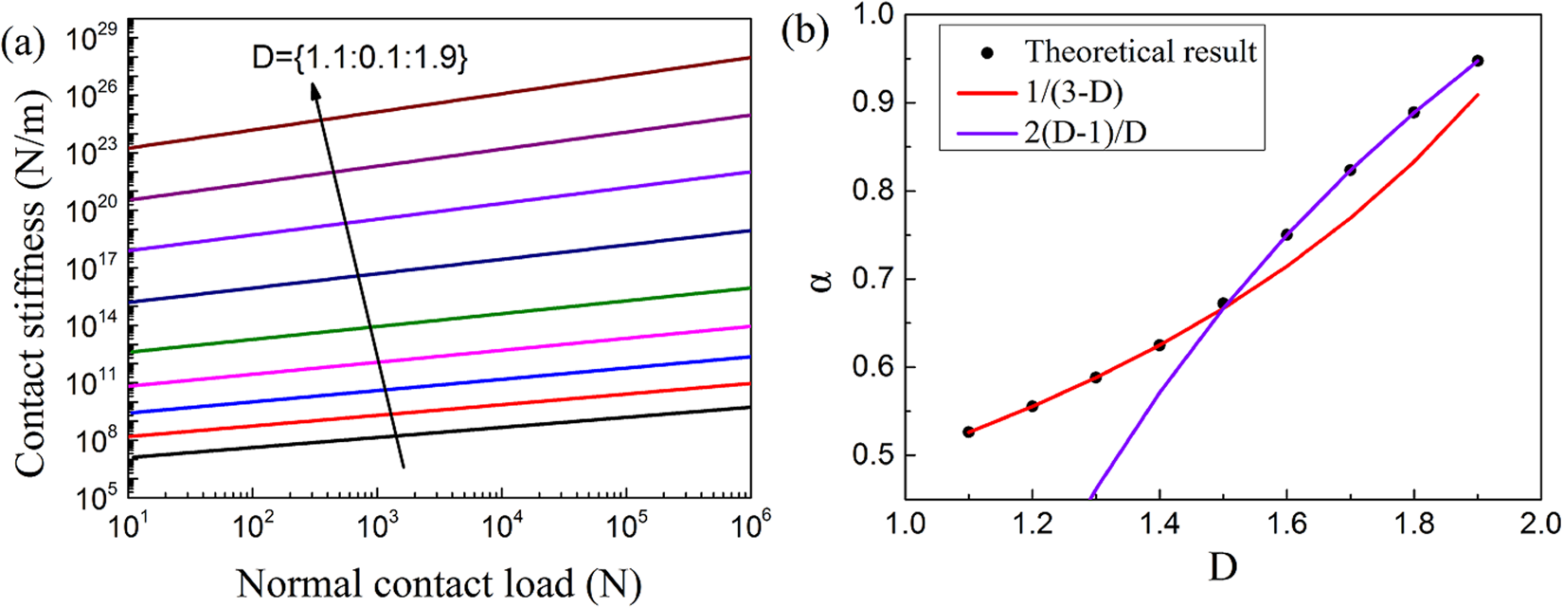
(**a**) Theoretical variations of the contact stiffness versus the normal load for different fractal dimensions when *G* = 1 × 10^−22^. (**b**) Dependency of the power *α* in the force-stiffness-relation versus *D*. The minimum coefficient of determination *R* ^2^ of the curve fitting within the 1.1 ≤ *D* ≤ 1.9 range is larger than 0.9999.

From the inspection of Fig. 3 one can also observe that the contact stiffness increases with the normal load by following a power law. This particular behavior contradicts the linear force-stiffness function predicted by the GW model, however it is more adequate to justify both the contact stiffness experimental measurements reported in^23^ and the boundary element simulations shown in^24^. Our theoretical model is able to illustrate the relation between contact stiffness and normal load for different fractal dimensions (Fig. 4). One can observe an evident log-log proportionality between stiffness and normal load within the force range of 10 N to 10^6^ N. The stiffness can be therefore described a power function of the normal load:

6 $${K}_{n}\propto {P}^{\alpha }$$

By curve fitting, the dependency of the power exponent *α* versus the fractal dimension *D* is illustrated in Fig. 4(b).

Pohrt and Popov^18^ have evaluated the contact stiffness of an elastic half-space and a rigid indenter with a randomly rough surface using the boundary element method, and concluded that when 0 < *H* < 2:

7 $$\alpha =\frac{1}{1+H}$$

within the range 0 < *H* < 1, the parameter *H* can be associated to the fractal dimension *D* ranging from 1 to 2 as:

8 $$D=2-H$$

Equation (8) leads to the conclusion that when $0 < D < 1$ the *α* exponent becomes:

9 $$\alpha =\frac{1}{3-D}$$

From Fig. 4(b) one can observe that the power exponent *α* predicted by our theoretical model follows almost exactly equation (9) when the fractal dimension is smaller than 1.5, and is slightly larger than the Pohrt and Popov predictions for higher fractal dimensions. The main reason behind this behavior is that all deformations have been assumed to be in the elastic range in^18^ (i.e., ${a}_{c}^{^{\prime} }=0$). When $1 < D < 1.5$ the exponent of ${a}_{c}^{^{\prime} }$ in equation (4) and the normal contact load are positive, and the hypothesis that ${a}_{c}^{^{\prime} }=0$ is therefore reasonable when the micro contact area is rather small compared to ${a}_{l}^{^{\prime} }$. In this case, the normal load and normal contact stiffness can be reduced as:

10 $$P\,\approx \,\frac{{2}^{(9-2D)/2}D{G}^{(D-1)}{E}^{\ast }\,{(ln\gamma )}^{1/2}}{3{\pi }^{(3-D)/2}(3-2D)}{a}_{l}^{^{\prime} (3/2-D/2)}$$

11 $${K}_{n}\,\approx \,\frac{4D(3-D){E}^{\ast }}{3\sqrt{2\pi }(3-2D)(2-D)}{({a}_{l}^{^{\prime} })}^{1/2}$$

The power relation in the force-stiffness function described by equations (10) and (11) is $1/(3-D)$ in this case.

When $1.5 < D < 2$, the exponent of the second term in the right hand part of equation (4) is negative. Since ${a}_{c}^{^{\prime} }$ is generally smaller than ${a}_{l}^{^{\prime} }$, the second term dominates both the normal contact load and contact stiffness. In this case:

12 $$P\,\approx \,-\frac{{2}^{(9-2D)/2}D{G}^{(D-1)}{E}^{\ast }{(ln\gamma )}^{1/2}}{3{\pi }^{(3-D)/2}(3-2D)}{a}_{c}^{^{\prime} (3/2-D)}{a}_{l}^{^{\prime} D/2}$$

13 $${K}_{n}\,\approx \,-\frac{4{D}^{2}{E}^{\ast }}{3\sqrt{2\pi }(3-2D)(2-D)}{a}_{c}^{^{\prime} (3/2-D)}{({a}_{l}^{^{\prime} })}^{(D-1)}$$

The power relation in the force-stiffness function described by equations (12) and (13) is $2(D-1)/D$, and corresponds to the simulation results shown in Fig. 4(b).

## Methods

### Theoretical model

The contact between two rough surfaces can be modeled as a single equivalent rugged surface in contact with a smooth rigid plane. The parameters *D* and *G* of the equivalent surface can be derived based on the profile distributions or structure functions of the two rugged surfaces^25^. The Young’s modulus of the equivalent contact surface material is calculated from:

14 $${E}^{\ast }\,=\,{[(1-{\nu }_{1}^{2})/{E}_{1}+(1-{\nu }_{2}^{2})/{E}_{2}]}^{-1}$$

where ${\nu }_{1}$, ${\nu }_{2}$, and ${E}_{1}$, ${E}_{2}$ are the Poisson’s ratios and Young’s moduli of the materials belonging to the original surfaces, respectively. The distribution function *n* of the truncated asperity sizes is mathematically similar to the one representing islands which spread over the surface of the Earth^26^, and can be written as:

15 $$n(a^{\prime} )\,=\,\frac{D}{2}\frac{{a}_{l}^{^{\prime} D/2}}{{a^{\prime} }^{(D/2+1)}}$$

The asperity interference $\delta$ ^17^ of a single asperity is determined by the fractal surface profile, and is equal to the peak-to-valley amplitude:

16 $$\delta \,=\,2{G}^{(D-1)}{(\mathrm{ln}\gamma )}^{1/2}{(2r^{\prime} )}^{(2-D)}$$

In equation (16) $r^{\prime}$ is the radius of the truncated micro-contact area $a^{\prime}$($a^{\prime} =\pi {r^{\prime} }^{2}$). The asperity interference is related to the radius of curvature *R* at the tip of the asperity by:

17 $${r^{\prime} }^{2}\,\approx \,2R\delta $$

By substituting equation (16) into (17), the radius of curvature $R$ is obtained as:

18 $$R\,=\,\frac{{a^{\prime} }^{D/2}}{{2}^{(4-D)}{\pi }^{D/2}{G}^{({\rm{D}}-1)}{(\mathrm{ln}\gamma )}^{1/2}}$$

Asperities with different truncated areas have different interferences and are subjected therefore to different deformation states. According to Greenwood and Williamson^12^, the critical value of the asperity interference at the onset of plastic flow is proportional to ${(H/{E}^{\ast })}^{2}R$. Consequently:

19 $${\delta }_{c}\,=\,b{(\frac{H}{{E}^{\ast }})}^{2}R$$

The parameter *b* is a constant independent of the asperity size, and *H* is the hardness of the softer material of two contacting surfaces. The deformation force of a single asperity is assumed to change continuously during the increase of the contact area, which results in:

20 $${P}^{e}({a}_{c}^{^{\prime} })\,=\,{P}^{p}({a}_{c}^{^{\prime} })$$

As a consequence of (20) we have $b=9{\pi }^{2}/4$. The critical truncated area can be derived by substituting equations (16)–(18) into equation (19):

In the simple case of elastic or fully plastic contact the asperities with $a^{\prime}  > {a}_{c}^{^{\prime} }$ are elastically deformed, while the ones with $a^{\prime} \le {a}_{c}^{^{\prime} }$ are in full plastic deformation. From Hertz contact theory the deformation force of an elastically deformed asperity is calculated as:

22 $${p}^{e}(a^{\prime} )=\frac{4{E}^{\ast }{r}^{3}}{3R}$$

where *r* is the radius of the micro-contact area *a* (with $a=a^{\prime} /2$). By substituting equation (18) into (22) the relationship between the deformation force and the truncated area can be derived as:

23 $${p}^{e}(a^{\prime} )=\frac{{2}^{(9-2D)/2}}{3{\pi }^{(3-D)/2}}{(ln\gamma )}^{1/2}{G}^{(D-1)}{E}^{\ast }{a^{\prime} }^{(3-D)/2}$$

The deformation force of a fully plastically deformed asperity is given by:

24 $${p}^{p}(a)=Ha^{\prime} $$

When ${a}_{l}^{^{\prime} }\le {a}_{c}^{^{\prime} }$ all the asperities belonging to an equivalent rough surface are deformed plastically, and the normal load of the contact surfaces can be calculated from integrating equation (24):

25 $$P={\int }_{{a^{\prime} }_{s}}^{{a^{\prime} }_{l}}{p}^{p}(a^{\prime} )n(a^{\prime} )da^{\prime} =\frac{HD}{2-D}{a}_{l}^{^{\prime} D/2}({a}_{l}^{^{\prime} (1-D/2)}-{a}_{s}^{^{\prime} (1-D/2)})$$

where ${a}_{s}^{^{\prime} }$ is the truncated area of the smallest asperity. Previous studies have shown that a surface can be fractal even at nanoscales^15^, which means typically that ${a}_{s}^{^{\prime} }$ is significantly smaller than ${a}_{l}^{^{\prime} }$ for realistic contacting surfaces. It is therefore valid to assume ${a}_{s}^{^{\prime} }\to {\rm{0}}$, and then:

26 $$P=\frac{HD}{2-D}{a}_{l}^{^{\prime} }$$

When ${a}_{l}^{^{\prime} } > {a}_{c}^{^{\prime} }$ both elastic and full plastic micro-contacts do exist. The normal contact load is therefore the sum of the integrals of equations (23) and (24):

27 $$P={\int }_{{a}_{s}^{^{\prime} }}^{{a}_{c}^{^{\prime} }}{p}^{p}(a^{\prime} )n(a^{\prime} )da^{\prime} +{\int }_{{a}_{c}^{^{\prime} }}^{{a}_{l}^{^{\prime} }}{p}^{e}(a^{\prime} )n(a^{\prime} )da^{\prime} $$

By inserting equation (15) into (27) and neglecting the influence of ${a}_{s}^{^{\prime} }$, the normal load in the case of ${a}_{l}^{^{\prime} } > {a}_{c}^{^{\prime} }$ is obtained as:

28 $$\begin{array}{rcl}P & = & \frac{{2}^{(9-2D)/2}D{G}^{(D-1)}{E}^{\ast }{(ln\gamma )}^{1/2}}{3{\pi }^{(3-D)/2}(3-2D)}({a}_{l}^{^{\prime} (3/2-D/2)}-{a}_{l}^{^{\prime} D/2}{a}_{c}^{^{\prime} (3/2-D)})\,\\  &  & +\frac{HD}{2-D}{a}_{l}^{^{\prime} D/2}{a}_{l}^{^{\prime} (1-D/2)}\,(1 < D < 2,D\ne 1.5)\\ P & = & 2{\pi }^{-3/4}{G}^{1/2}{E}^{\ast }{(\mathrm{ln}\gamma )}^{1/2}{a}_{l}^{^{\prime} 3/4}\,\mathrm{ln}({a}_{l}^{^{\prime} }/{a}_{c}^{^{\prime} })+3H{a}_{l}^{^{\prime} 3/4}{a}_{c}^{^{\prime} 1/4}\,(D=1.5)\end{array}$$

It can be deduced from equation (16) that the asperity interference is directly proportional to the truncated micro-contact area. As a consequence, the largest interference is represented by the interference of the asperity with the largest truncated area:

29 $${\delta }_{\max }={2}^{(3-D)}{G}^{(D-1)}{(\mathrm{ln}\gamma )}^{1/2}{\pi }^{(D-2)/2}{({a}_{l}^{^{\prime} })}^{(2-D)/2}$$

Equations (26), (28) and (29) show that the normal load and the relative displacement of the rigid plane from the equivalent rough surface are both functions of ${a}_{l}^{^{\prime} }$. Therefore, the normal contact stiffness can be calculated as:

30 $${K}_{n}=\frac{dP}{d{a}_{l}^{^{\prime} }}{(\frac{d{\rm{\Delta }}}{d{a}_{l}^{^{\prime} }})}^{-1}$$

### Experiment

**Figure 5:**
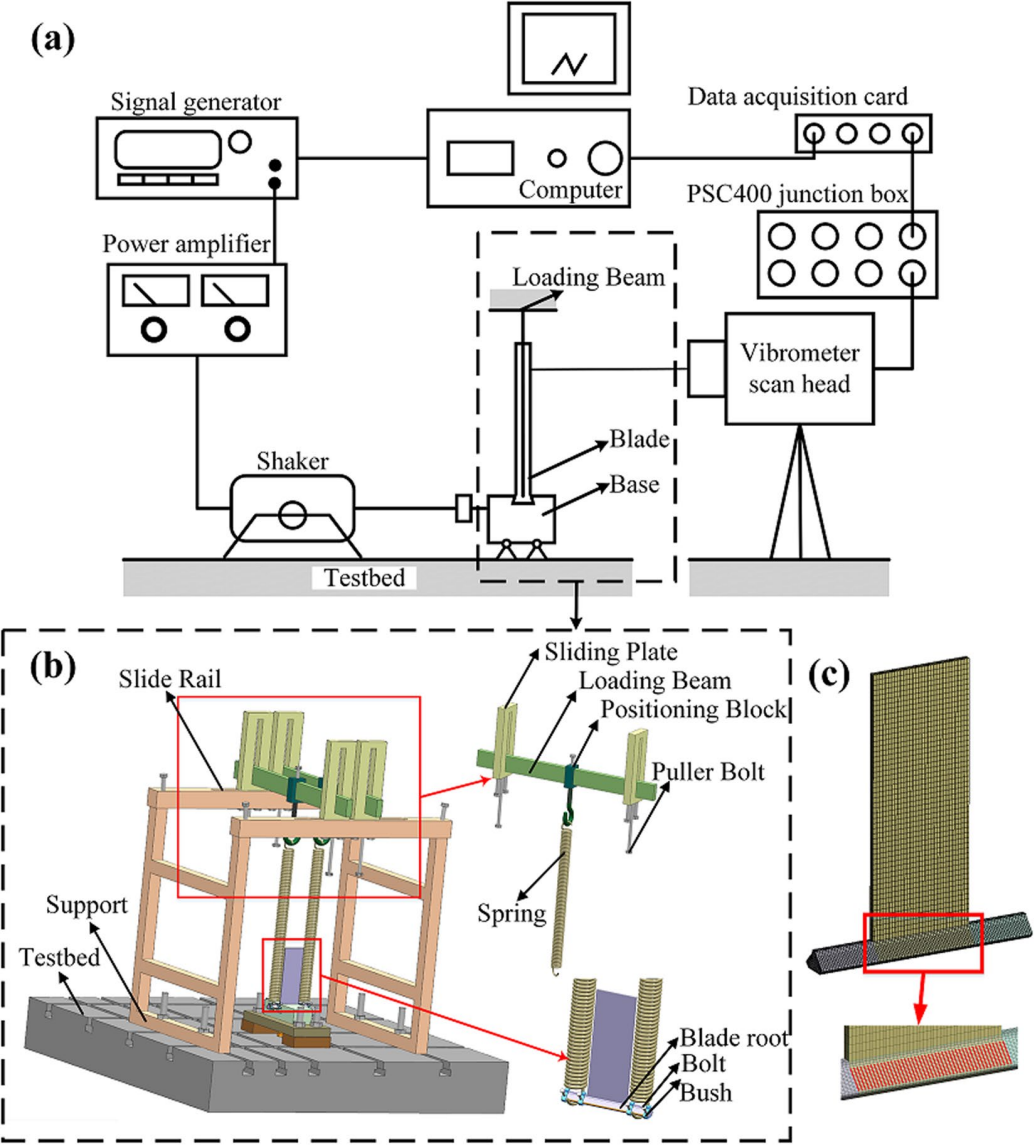
(**a**) Sketch of the measurement system, (**b**) the loading device to tune the normal contact load on the dovetail joint, and **(c)** the finite element model of the blade.

Figure 5 shows a diagram illustrating the whole measurement system. The test rig is a dovetail joint formed by a planar blade inserted into a slot carved in a base. The blade inside the slot represents a portion of a bladed disk. There are two symmetric contact interfaces, whose 90 ° angle is determined by the geometry of the dovetail. The contact interfaces are produced by milling the material (No. 45 steel with ${\rm{E}}=\mathrm{201\; GPa},\,{\rm{\nu }}=0\mathrm{.30\; and\; HB}=\mathrm{200}$)^27^. The fractal parameters *D* and *G* are determined with reference to the equivalent parameter values of the contact pairs machined by milling^16^, which is $D=1.2183,G=5.9036E-14$.

A loading device is custom designed to facilitate the fine adjustment of the load to the blade and the normal load on the dovetail joint. The dovetail root of the blade is designed to extend beyond the ends, and two springs restricted by four bushes apply the load by simulating a centrifugal force (see Fig. 5 (b)). Springs exert nearly no influence on the dynamic characteristics of the system, and this provides benefits to type of measurement performed. The four sliding plates that guide the loading beams can freely and independently move along the corresponding rails of the support. The position blocks connected to the springs can also freely move on the loading beams. In this way it is possible to adjust the position of the sliding plates and the positioning blocks to guarantee that the applied tension is vertical. The fine tuning of the tension is achieved by screwing the four puller bolts. The value of the load can be easily and accurately obtained from measuring the deformation of the springs, each having a nominal stiffness of 6.9 N∙mm^−1^. The force-displacement curves of the two springs are measured by compressive tests using a WDW 3100 electronic universal testing machine, with a 1 kN force sensor (accuracy of 4‰) and a 25 mm electronic dial gauge (accuracy of ± 0.005 mm). The strain rate is 0.1 mm∙s^−1^. The results show the two test springs are linear (average stiffness of 6.89 N∙mm^−1^) and coincident with each other, and are therefore capable to apply the load to the blade in a uniform manner. The range of the applied load to blade is chosen to be within 0 N ~ 1,400 N to ensure a noticeable change of the resonant frequency when the applied load is varied. Given that the interfacial frictional force is actually varying during the vibration, the average frictional force is used and thus the relationship between the applied load to the blade and the normal contact load at one side is:

31 $$P=\frac{F}{2\,\cos \,\theta (1+\bar{\mu }\,\tan \,\theta )}$$

where *F* is the applied load, *P* is the normal contact load of one side, *θ* is the dovetail angle with *θ* = 90° and *μ* is the nominal friction coefficient with $\mu =0.2$ ^28^

When the magnitude of the applied load is relatively low the blade has an easy relative motion with the slot. In a traditional full contact arrangement between the pushrod of the shaker and the loosely restricted blade, the additional mass and stiffness effect provided by the rod would have a significant influence on the dynamic characteristics of the system, and this is unacceptable for the type of measurement performed here. We use instead a base excitation method in which the shaker applies the dynamic load to the base (Fig. 5 (a)). Based on preliminary trials the sweep frequency range is between 115 Hz and 138 Hz, with a sine sweep time of 200 s to guarantee the high accuracy of the mode test. The excitation voltage applied to the shaker is also as small as possible to minimize the influence of relative slip of the surfaces. As a consequence, the laser spot to measure the dynamic structural velocity is located near the tip of the blade. The excitation voltage for the electrodynamic shaker is kept at 0.14 V during the whole experiment. To check the repeatability of the test rig, the frequency response functions were measured with springs extended to 28 mm and unloaded five times. The results show that the maximum error between the five resonant frequencies is only 0.1%.

### Identification of the contact stiffness

The identification of the contact stiffness is based on the contact resonant frequency method^29,30^ which has been widely used to indirectly measure the interfacial contact stiffness. Typically, the system resonant frequency is constant and independent of the load, whereas the contact resonant frequency will shift with increasing contact load because both interfacial stiffness and damping change. The damping effect can be ignored in the calculation, since the resonant frequency of the FRF with reference to the velocity response is equal to the natural frequency of the system. An undamped modal analysis is therefore sufficient to determine the resonant frequency.

The finite element model used for the inverse identification of the test planar blade is shown in Fig. 5 (c). The model was developed using the commercial code ANSYS 17.0. The element type is SOLID 186, while the element size is overall between 1.2 mm and 1.5 mm with 117,599 nodes in total. The influence of the contact interfaces on the dynamic characteristics of the blade is simulated by adding springs to nodes located on the interfaces (the red spots in Fig. 5(c)). If we suppose that the contact stiffness is uniformly distributed and ignore the effect provided by partial slip, the normal and tangent contact stiffness of each marked node (*k*_*ni*_ and *k*_*ti*_, respectively) can be calculated as:

32 $${k}_{ni}=\frac{{K}_{n}}{N}$$

33 $${k}_{ti}=\frac{2(1-\nu )}{(2-\nu )}{k}_{ni}$$

where *N* is the number of nodes on one interface, *i* is the node number.

The above stiffness is defined by spring elements (COMBINE 14) on each marked node in the finite element model. The 1^st^ natural frequency corresponding to any given contact stiffness can be obtained by performing an eigenvalue modal analysis with block Lanczos method. We can then obtain the relationship between the interfacial contact stiffness *K*_*n*_ and the measured resonant frequency according to the FEA results. Eventually the contact stiffness can be inversely identified by fitting the simulation results to the measured 1^st^ resonant frequency.

### Data availability

All data generated or analysed during this study are included in this published article.

## Conclusions

One generalized model of the interfacial contact stiffness between fractal rough surfaces is developed by taking into account the effect of both elastic and plastic deformations of asperities. It can be inferred from the model that typically the contact stiffness is a power law function of the normal contact load with an exponent *α* within the whole range of fractal dimension *D*(1 < *D* < 2). For 1 < *D* < 1.5 the Pohrt-Popov behavior ($\alpha =1/(3-D)$) is valid, however for 1.5 < *D* < 2, the exponent *α* is slightly larger and equal to $2(D-1)/D$. Moreover, the model is validated by the experimental results obtained from an original test rig which simulates the dovetail joints for turbo machinery blades. The model exhibits excellent precision and better universality than the Pohrt-Popov one, and it is valuable for the research on static and dynamic characteristics of any mechanical systems where unlubricated contacting surfaces with fractal roughness present.
